# Partial deletion of distal long arm encompassing Jacobsen Syndrome

**DOI:** 10.1186/1755-8166-7-S1-P47

**Published:** 2014-01-21

**Authors:** Manisha Desai, Bhumi Patel, Chaitanya Datar, Anand Pandit, Prakash Ghambhir, Darshana Nayak, Jayesh Sheth, Frenny Sheth

**Affiliations:** 1 FRIGE’s Institute of Human Genetics, FRIGE House, Jodhpur Gam Road, Satellite, Ahmedaba, India; 2 Sahyadri Genetics, Unit of Sahyadri Hospitals, Barve Memorial Complex, J.M. Road, Pune, India; 3 KEM Hospital, Department of Paediatrics, Rasta Peth, Pune, India; 4 Birth Right Genetic Clinic, Erandawane, Pune, India; 5 Asian Child Neuro Clinics, Park Avenue, Ellisbridge, Ahmedabad, India

**Keywords:** Jacobsen syndrome, 11q deletion, array-CGH, quantitative Genomic imbalance, inheritance

## Background

The terminal 11q deletion syndrome also known as Jacobsen syndrome (JS) is a rare genetic disorder associated with multiple dysmorphic features and occurs in 1 in 100,000 live births. The etiology behind the disorder is loss of contiguous set of genes due to 7-20Mb deletion that has proximal breakpoint at 11q23.

## Materials and methods

Segmental aneuploidy or alteration involving #11q arm was detected in 2 cases during conventional cytogenetic analysis carried out in children having multiple congenital anomalies (MCA). Chromosome preparations were obtained from PHA stimulated lymphocyte cultures according to the standard procedure at 500-band level in both patient and their parents. Evaluation of the break point region was performed by 60K oligonucleotide array-Comparative Genomic Hybridization (aCGH) using Agilent platform. Female genomic DNA (Promega Corporation, Madison, WI, USA) was used as a sex-matched reference, which was analyzed with the aCGH analysis software v3.4 (Agilent Technologies Inc., Santa Clara, CA, USA) by applying Z-score segmentation algorithm with a window size of 10 points to identify chromosome aberrations.

## Results

Chromosomal study demonstrated structural rearrangements on #11q arm in both the cases i.e. 46,XX,der(11)del(11)(q24). Oligonucelotide aCGH analysis was performed using 3-points filter and 0.2 variation which lead to the confirmation of partial deletion of 11.8-11.9Mb at 11q24.1q25 [arr 11q24.1q25(123,045,174-134,868,407)x1] in case-1 where presence of deletion was suggested by banding technique. Whereas, a 13.9-14Mb deletion at 11q23.3q25 together with 7.3-7.6Mb duplication at 12q24.32q24.33 was detected in the second case [arr 11q23.3q25(121,000,318-134,868,407)x1 and 12q24.32q24.33(126,482,698-133,767,986)x3]. The complex structural rearrangement was missed by the conventional cytogenetic analysis in case 2. Paternal inheritance was confirmed in the latter case.

## Conclusion

Conventional cytogenetic analysis provided an overview of the deleted segment whereas aCGH analysis added detailed information about the breakpoint region. The complex structural rearrangement detected only by aCGH indicates its utility in diagnosis of rare genetic diseases especially in cases with multiple congenital anomalies.

